# Glutamate and GABA Homeostasis and Neurometabolism in Major Depressive Disorder

**DOI:** 10.3389/fpsyt.2021.637863

**Published:** 2021-04-27

**Authors:** Ajay Sarawagi, Narayan Datt Soni, Anant Bahadur Patel

**Affiliations:** ^1^NMR Microimaging and Spectroscopy, CSIR-Centre for Cellular and Molecular Biology, Hyderabad, India; ^2^Academy of Scientific and Innovative Research, Ghaziabad, India

**Keywords:** antidepressant, brain, ^13^C-NMR spectroscopy, glutamine, ketamine, neurocircuitry, neurometabolism, neurotransmitter

## Abstract

Major depressive disorder (MDD) is a leading cause of distress, disability, and suicides. As per the latest WHO report, MDD affects more than 260 million people worldwide. Despite decades of research, the underlying etiology of depression is not fully understood. Glutamate and γ-aminobutyric acid (GABA) are the major excitatory and inhibitory neurotransmitters, respectively, in the matured central nervous system. Imbalance in the levels of these neurotransmitters has been implicated in different neurological and psychiatric disorders including MDD. ^1^H nuclear magnetic resonance (NMR) spectroscopy is a powerful non-invasive method to study neurometabolites homeostasis *in vivo*. Additionally, ^13^C-NMR spectroscopy together with an intravenous administration of non-radioactive ^13^C-labeled glucose or acetate provides a measure of neural functions. In this review, we provide an overview of NMR-based measurements of glutamate and GABA homeostasis, neurometabolic activity, and neurotransmitter cycling in MDD. Finally, we highlight the impact of recent advancements in treatment strategies against a depressive disorder that target glutamate and GABA pathways in the brain.

## Introduction

Major depressive disorder (MDD) is a neuropsychiatric condition, characterized by low mood, loss of interest in pleasurable activities, and suicidal ideation. It affects ~5% of the population worldwide (1). As per the WHO report (2020), around 0.8 million people commit suicide every year, and more than 90% of these had a psychiatric diagnosis (2, 3). MDD is one of the major contributors to chronic disease burden over the world population, and imparts a high socioeconomic impact (4). Despite several decades of research, there are no robust physiological and molecular markers for psychiatric disorders. Therefore, diagnosis of these disorders is achieved mostly by questionnaire-based psychiatric evaluation. The diagnostic criteria for psychiatric disorders have been evolving continuously. The diagnostic standards and specifiers of MDD as per the latest edition of the Diagnostic and Statistical Manual of Mental Disorders (DSM-5, 2013) are described in Box 1 (5). Very often, the symptoms of different neuropsychiatric disorders overlap with each other and interfere in precise diagnosis. Hence, there is a need for extensive research on the identification of biomarkers for the development of novel diagnostic strategies for MDD. Depression is a highly variable disorder with multiple risk factors and causes that vary at the individual level. Certain environmental factors such as prematernal stress, childhood abuse, physical and sexual abuse, continuous failures, substance abuse, sadness and severe trauma increase the risk of depression (6, 7). Depression has been often seen to be associated with various neurodegenerative disorders (8) such as Parkinson's disease, Alzheimer's disease, amyotrophic lateral sclerosis, and systemic diseases like diabetes (9) and cancer (10).

Box 1 **Symptoms of MDD: as per diagnostic and statistical manual of mental disorder (DSM-V) at least five of the following symptoms must be present during entire 2-week period (5).**

➢ Consistently feeling sad, empty, and hopeless
➢ Markedly diminished interest in pleasurable activities
➢ Significant weight loss or weight gain
➢ Increased or decreased appetite
➢ Insomnia or hypersomnia
➢ Fatigue or loss of energy
➢ Feeling of worthlessness, feeling excessive, or inappropriate guilt
➢ Diminished ability to think or concentrate, or indecisiveness
➢ Recurrent thoughts of death and suicidal ideation without a specific plan
➢ Psychomotor agitation or retardation

These symptoms cause clinically significant distress or impairment in social, occupational, or other important areas of functioning. Moreover, the episode is not attributable to the physiological effects of a substance or another medical condition.

**Figure 1:**
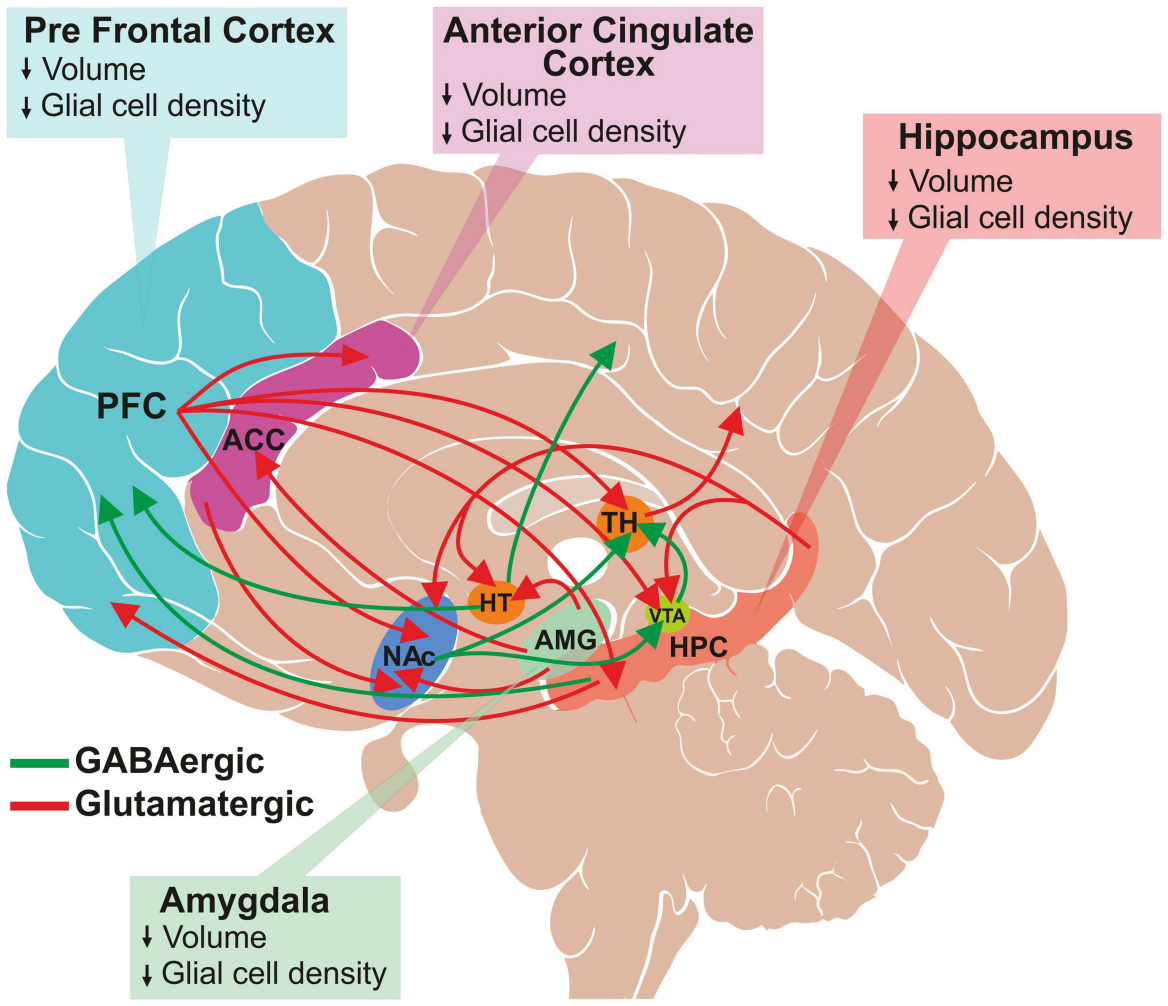
Schematic of glutamatergic and GABAergic projections involved in mood regulation and reward pathway. A subset of several known interconnections among different brain regions are shown. Major glutamatergic projections (red color) arise from the frontal cortex to the anterior cingulate cortex (ACC), thalamus (TH), ventral tegmental area (VTA), hippocampus (HPC) and nucleus accumbens (NAc). Additionally, glutamatergic neurons originate from hippocampus, and innervate into hypothalamus (HT), VTA, NAc and PFC and from amygdala to HT, ACC and NAc. The GABAergic projections (green color) are widely distributed throughout the brain. The major projections that are relevant to this review are from HT to the occipital and parietal cortex, HPC to PFC, and NAc to the thalamus and VTA. The structural changes observed in the brain regions of depressed subjects are shown in the respective boxes.

Despite enormous efforts made by the global psychiatric research community, the molecular mechanism of MDD is not yet very clear. Several neuroimaging and postmortem studies have shown a loss of neuronal and glial population in the cingulate cortex, prefrontal cortex (PFC) (11) and hippocampus (12, 13) of depressed subjects (Figure 1) (14, 15). Various genetic factors (16), epigenetic changes (17) and endocrine pathways (18) are believed to be involved in the pathophysiology of the disorder. The elevated activity of the hypothalamic–pituitary–adrenal (HPA) axis is at the heart of the neurobiological presumptions of depression (19). Higher activity of HPA axis increases levels of glucocorticoids in blood, plasma and cerebrospinal fluid (CSF), which are greatly associated with stress. Additionally, environmental factors and stress influence neuronal function epigenetically. These factors alter gene expression by histone acetylation or DNA methylation (20).

The role of epigenetics in depression is supported by studies reporting antidepressive effects of histone deacetylase inhibitors in rodent models of depression (20, 21). Additionally, a large number of studies have reported a reduced level of brain-derived neurotrophic factor (BDNF) in the hippocampus (HPC) and PFC of depressed subjects (22). BDNF is crucial for the activity-dependent formation and maintenance of synapses by regulating the activity of the mTORC1 complex. Activation of mTORC1 pathways promotes *de novo* synthesis of various synaptic proteins, including GluA1, α-amino-3-hydroxy-5-methyl-4-isoxazolepropionic acid (AMPA) receptor subunits and postsynaptic density protein 95 (PSD95) (23). Interventions with different antidepressants have shown increased expression of BDNF in PFC of the rodent brain (24, 25).

Neurotransmitters are the chemical messengers present in presynaptic nerve terminals and are released into the synaptic cleft in response to the action potential (26). These neurotransmitters bind to specific receptors present on the postsynaptic membrane, and thus facilitate the transmission of the action potential across the synapse (26). Neurotransmitters are broadly classified into amino acids, peptides, and monoamines depending on their chemical properties. Amino acid neurotransmitters include glutamate, γ-aminobutyric acid (GABA), aspartate and glycine, which are abundant in the central nervous system (CNS). Substance P, cholecystokinin, opioids and neuropeptide Y belong to the peptide neurotransmitter category (27). In the monoamine category, several neurotransmitters including serotonin, dopamine, norepinephrine and epinephrine are well-studied, and are shown to be involved in various neuropsychiatric disorders (28, 29). Functionally, glutamate, aspartate, dopamine, epinephrine and norepinephrine are considered as excitatory neurotransmitters, while GABA, glycine and serotonin are the major inhibitory neurotransmitters in the matured mammalian CNS (30).

^1^H magnetic resonance spectroscopy (MRS) has emerged as a powerful non-invasive method for the measurement of levels of neurometabolites including glutamate and GABA in the brain (31). In addition, ^13^C-MRS in conjunction with administration of ^13^C-labeled respiratory substrates (glucose and acetate) allows analysis of the cell-specific metabolic activity in animals as well as in the human brain. This provides a non-invasive measurement of the cerebral metabolic rate of glucose oxidation, ATP production and neurotransmitter cycling (32, 33).

### Neurocircuitry of Reward and Emotions

Depression is characterized by a deficit in various aspects of reward, which is defined as responses toward positive emotional stimuli such as food, sex and social interaction (34). Several brain regions such as prefrontal cortex (PFC), nucleus accumbens (NAc), ventral tegmental area (VTA), hippocampus (HPC) and amygdala are interconnected with each other via dopaminergic, serotonergic, glutamatergic and GABAergic neurons, which comprise the reward circuit (35, 36). The reward circuitry mainly includes dopaminergic projection from VTA to NAc, PFC, hippocampus, amygdala, as well as other brain regions. Additionally, glutamatergic and GABAergic projections interconnect these regions very densely (Figure 1). The cortical glutamate connections can be divided broadly into five major arcs that include PFC to the brainstem, PFC to the striatum and NAc, cerebral cortex to the thalamus, intracortical glutamate projections, and from the thalamus to the cerebral cortex (37, 38). Moreover, glutamatergic connections are found in subcortical regions: hippocampus to VTA, hypothalamus, NAc and PFC; and amygdala to NAc, hypothalamus and ACC. GABAergic neurons also make dense connections between brain regions that include projections from the striatum to substantia nigra (SN) and brainstem; thalamus to SN; HPC to occipital and parietal cortex; HPC to thalamus and striatum; NAc to VTA and thalamus; and VTA to PFC and NAc (Figure 1) (37, 39). The brain reward regions have been linked with specific behavioral functions, e.g., PFC for decision making and intelligence, HPC for emotional management, amygdala as the fear center, and NAc-VTA for motivation, pleasure and reward. These brain regions have broader functions in the management of emotional and cognitive behavior. Various imaging and postmortem studies have shown reduced volume and atrophy in these brain regions of depressed subjects and animal models of depression (11–15).

### Neurometabolites Homeostasis in Healthy Brain

Neurometabolites homeostasis plays a very important role in brain function, and has been shown to be affected in animal models and human subjects of various neuropsychiatric disorders including MDD (40, 41). Several small molecules including N-acetyl-aspartate (NAA) (~9 μmol/g), alanine (~1 μmol/g), aspartate (~1.2 μmol/g), choline (~1.5 μmol/g), creatine (~7 μmol/g), GABA (~1.5 μmol/g), glutamate (~10 μmol/g), glutamine (~2.5 μmol/g), glycine (~1 μmol/g), myo-inositol (~6 μmol/g) and taurine (~1.2 μmol/g) contribute to major fraction of neurometabolites pool in healthy brain (42).

**Figure 2:**
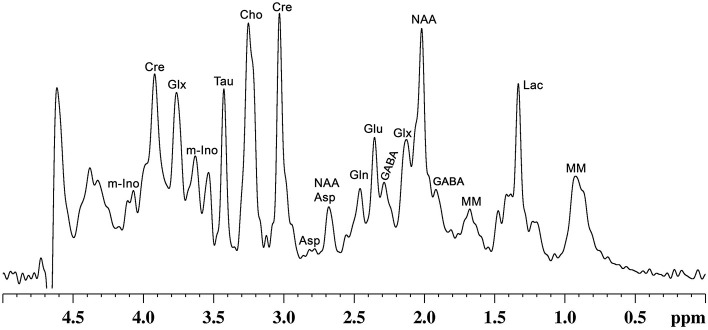
A representative localized *in vivo* ^1^H-NMR spectrum from mouse cerebral cortex. NMR spectrum was recorded using, a vertical wide bore magnet interfaced with 600 MHz MR spectrometer. ^1^H-MR spectroscopy was carried out using STEAM method in conjunction with outer volume suppression (OVS) and water suppression (VAPOR) from a voxel (4.0 × 1.2 × 2.5 mm^3^) with TE/TR = 4/4,000 ms with 512 averaging: Peak labels are Asp, aspartate; Cho, choline; Cr, creatine; GABA, γ-aminobutyric acid; Gln, glutamine; Glu, glutamate; Glx, glutamate + glutamine; Lac, lactate; m-Ino, myo-inositol; MM, macromolecule; NAA, N-acetyl aspartate; Tau, taurine.

In addition to precursors for different metabolites, these molecules play various critical functions that include signal transduction, osmoregulation, cell growth and protein synthesis (42). Glutamate released into synaptic cleft increases the membrane potential of postsynaptic neurons, making them more likely to lead to an action potential. Moreover, it plays a critical role in long-term potentiation (43), synaptic plasticity (44), learning and memory (45), and various cognitive functions (46). Likewise, GABA, the major inhibitory neurotransmitter in the matured CNS, inhibits the propagation of action potential (47). Several studies have revealed the involvement of GABA in learning and memory (48–50), aggressive–defensive behavior, and impulsivity (51, 52). NAA is localized mostly in neurons, and is known to be a marker of neuronal viability and health. It acts as a precursor for NAAG, the storage form of aspartate, and serves a variety of other functions (53). Myo-inositol, mostly localized in astroglia, acts as an osmolite, plays an essential role in cell growth, and is believed to be a marker of the glial population (42). Moreover, it is considered an inflammatory marker in CNS (42). Nearly 20 vital metabolites, which include the above-mentioned molecules, can be detected and quantified *in vivo* by different MR spectroscopic approaches in human (54) and animal brains (55) (Figure 2). The most commonly used NMR methods for detection and quantification of brain metabolites are described in the subsequent section.

### Glutamate and GABA Energy Metabolism in Brain

**Figure 3:**
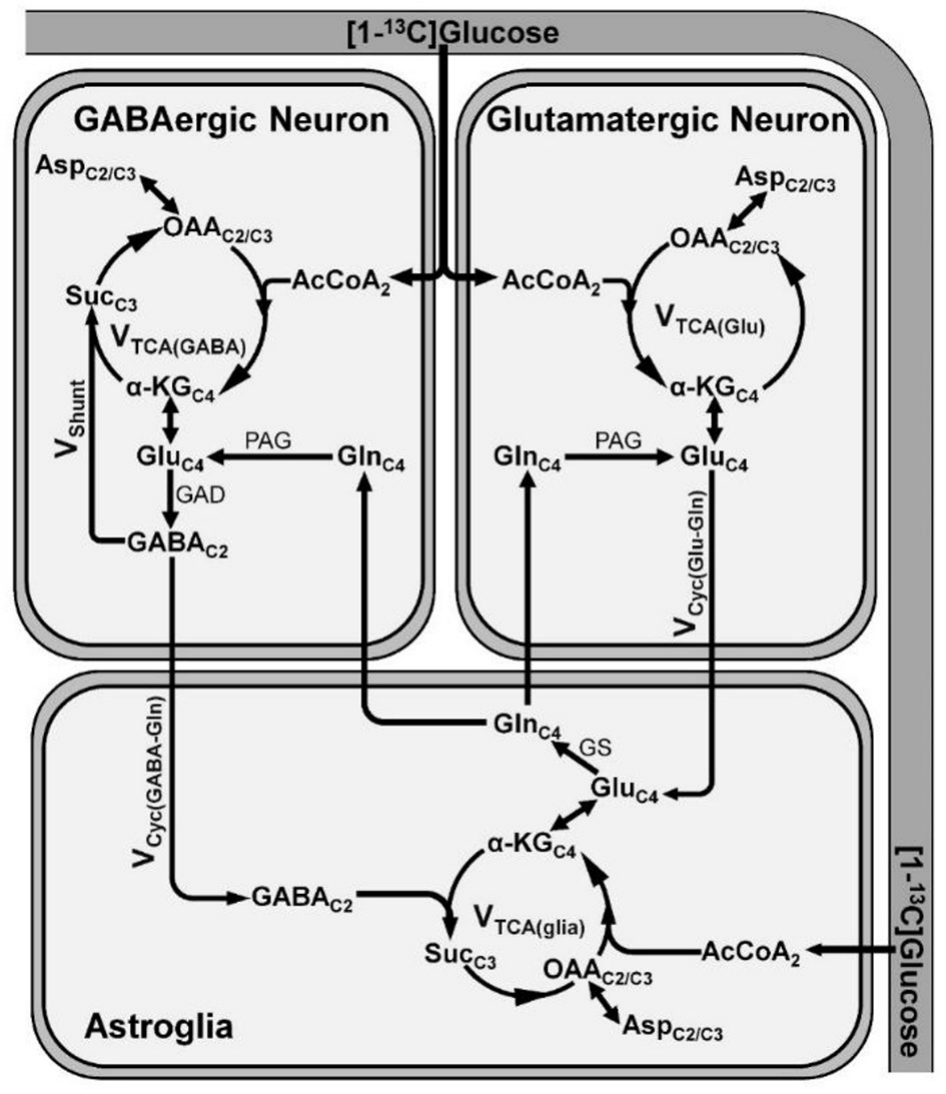
A schematic of three compartment metabolic model showing ^13^C labeling of amino acids from [1-^13^C]glucose. Metabolism of [1-^13^C]glucose via glutamatergic and GABAergic TCA cycle labels Glu_C4_. In GABAergic neurons, Glu_C4_ is further decarboxylated to GABA_C2_ by glutamate decarboxylase (GAD). The labeling of Gln_C4_ occurs by release and uptake of Glu_C4_ and GABA_C2_ in astrocytes followed by transamination by glutamine synthetase (GS). Further metabolism of Glu_C4_ and GABA_C2_ transfers the label into Asp_C2/C3_. α-KG_C4_, α-ketoglutarate-C4; AcCoA_2_, acetyl co-enzymeA-C2; Asp_C2/C3_, aspartate-C2/C3; GABA_C2_, γ-aminobutyric acid-C2; Glu_C4_, glutamate-C4; GAD, glutamate decarboxylase; Gln_C4_, glutamine-C4; GS, glutamine synthetase; OAA_C2/C3_, oxaloacetate-C2/C3; PAG, phosphate activated glutaminase; Suc_C3_, succinate-C3; V_cyc(GABA−Gln)_, GABA–glutamine cycling flux; V_cyc(Glu−Gln)_, glutamate–glutamine cycling flux; V_shunt_, flux of GABA shunt; V_TCA(glia)_, astroglial TCA cycle flux; V_TCA(GABA)_, GABAergic TCA cycle flux; V_TCA(Glu)_, glutamatergic TCA cycle flux.

The human brain accounts for 2% of the body weight, but it contributes to 20% of the total energy consumed, indicating the overwhelming energy demand of the brain (56, 57). In a matured brain, this energy requirement is majorly fulfilled by the oxidation of glucose. Most of the energy harvested in the brain is utilized for the processes associated with glutamatergic and GABAergic neurotransmission (57). The glutamate released from glutamatergic neurons into the synaptic cleft is taken up by astrocytes and converted to glutamine by glutamine synthetase. Glutamine is transported back to neurons, hydrolyzed to glutamate, and repackaged into vesicles for the next release. This process is referred as glutamate–glutamine neurotransmitter cycling (58). Similarly, substrate cycle involving GABA and glutamine (GABA–glutamine) occurs between GABAergic neurons and astrocytes (58). In this cycle, the released GABA into the synapse is taken up majorly by astrocytes, wherein it is metabolized to succinate by GABA-transaminase, and enters into the TCA cycle and ultimately converted to glutamine. The glutamine thus formed is further transported to GABAergic neurons and converted to GABA by the successive action of glutaminase and glutamate decarboxylase (59, 60). The rates of neuronal glucose oxidation and neurotransmitter cycling have been monitored by a tracer approach, wherein ^13^C-labeled glucose is administered intravenously, and labeling of brain amino acids is measured *in vivo* by ^13^C-NMR spectroscopy (61). The metabolism of [1,6-^13^C_2_]glucose via glycolysis followed by TCA cycle labels Glu_C4_ in glutamatergic and GABAergic neurons (Figure 3). In GABAergic neurons, Glu_C4_ is decarboxylated to GABA_C2_ by glutamate decarboxylase (GAD). Gln_C4_ gets labeled from Glu_C4_ and GABA_C2_ through glutamate–glutamine and GABA-glutamine neurotransmitter cycling, respectively. Further metabolism of Glu_C4_ and GABA_C2_ in the corresponding TCA cycle labels Asp_C2/C3_, Glu_C2/C3_, and GABA_C3/C4_. The kinetics of label incorporation in different amino acids is analyzed to determine the rate of glucose oxidation in the glutamatergic, GABAergic neurons, and rate of neurotransmitter cycling (59). Energy budget estimates for the cost of signaling based on anatomic and physiological data in the cerebral cortex indicated that most of the signaling energy is utilized on postsynaptic glutamate receptors, followed by action potentials and resting potentials. In the cerebellar cortex, glutamatergic neurons use 75%, while GABAergic neurons use 25% of the signaling energy (57). Hence, an estimate of the energy expenditure of the glutamatergic and GABAergic neurons using ^13^C-MRS approach directly reflects their functional status.

## Hypothesis

The most prevalent hypothesis of depression posits that depletion in monoamine neurotransmitters level is the underlying cause of the disease (62, 63). Recent studies in animal models and human subjects have suggested an association of glutamatergic and GABAergic systems with the pathophysiology of depression (64–66). Reduced expression of receptor subunits, imbalances in their levels, decreased glutamatergic and GABAergic neurotransmission, and altered energy metabolism are known to play a critical role in the progression of depression (64, 65).

### Glutamatergic Hypothesis of Depression

Glutamatergic neurons constitute approximately 80% of the synapses in the neocortex (67). Glutamate is released at synapses throughout the brain, and exerts changes in postsynaptic excitability and neuroplasticity (68). It activates various downstream pathways of nuclear genes by binding to a variety of membrane-bound receptors present on the postsynaptic membrane, which regulate secondary messenger systems. α-Amino-3-hydroxy-5-methyl-4-isoxazolepropionic acid (AMPA_R_), N-methyl-D-aspartate (NMDA_R_), and kainate are the fast-acting ionotropic receptors that get activated by glutamate binding (69). Glutamate also binds to G-protein-coupled receptors, known as metabotropic glutamate receptors, which mediate various cellular processes and slow-acting changes through secondary messengers such as cyclic adenosine monophosphate (cAMP), cyclic guanosine monophosphate (cGMP) and phosphatidylinositol (69). AMPA and kainate receptors help in the conduction of action potential primarily through the flux of Na^+^ ions, while NMDA_R_ is distinguished by its more permeability to Ca^2+^ ions. NMDA receptor signaling promotes various responses such as excitation, neurotrophic function, and can even activate cell death pathways. Abnormal activity of NMDA receptor imparts harmful effects on neurons (69). Overexcitation of NMDA_R_ by excessive glutamate release or impaired synaptic clearance leads to the death of neurons by excitotoxicity (70).

A large number of clinical as well as animal studies have reported impairment in the glutamatergic system in various limbic and cortical areas of the brain of depressed subjects (71, 72). Additionally, postmortem histopathology (73) and a number of ^1^H-MRS studies (74, 75) have shed light on the association of the aberrant glutamate system with maladaptive changes in the structure and function of excitatory circuitry. Several studies have reported decreased expression of NMDA (73, 76, 77) and AMPA receptor subunits (77, 78) in PFC of depressed individuals. Reduced expression of NMDA receptor subunits has also been seen in the postmortem brains of suicide victims (73, 79). Moreover, the decreased availability of metabotropic receptor mGluR5 in PFC, cingulate cortex, thalamus, hippocampus, and other cortical regions has been reported in depressed individuals (80, 81). Additionally, loss of glutamatergic neurons in the orbitofrontal cortex is associated with the pathophysiology of depression (82). These shreds of evidence suggest the involvement of glutamatergic system with the pathophysiology of MDD.

### GABAergic Hypothesis of Depression

Glutamate acts as the precursor for GABA, the predominant inhibitory neurotransmitter in the matured brain (83). GABAergic neurons contribute to one-third of total synapses in the CNS and help in shaping the neural network dynamics (84). These inhibitory neurons are known to play a pivotal role in physiological processes that are often affected in psychiatric disorders such as neural plasticity, sensory processing, stress reactivity, memory formation, and attention (84, 85). GABA binds to two different classes of receptors, the fast-acting ligand gated or ionotropic receptor GABA_A_ and GABA_B_. Activation of GABA_A_ receptor leads to an influx of chloride ions, which inhibits the propagation of action potential. However, activation of GABA_B_ receptors stimulates K^+^ channel opening, which helps in achieving a hyperpolarized state that leads to reduced transmission of action potential (86, 87).

GABAergic interneurons are identified by their expression of specific receptors for somatostatin (SST), parvalbumin (PV), and 5-HT3a. SST and PV interneurons make up to 30 and 40%, respectively, of the total GABAergic neuronal pool (88). Postmortem studies of depressed subjects have shown a reduced level of SST and PV interneurons in PFC as well as in other cortical areas (89). Additionally, a decrease in the level of SST messenger RNA (mRNA) has been reported in several brain regions, including dorsolateral PFC (90, 91), ACC (92) and amygdala (93) in depression (47). Moreover, multiple studies have reported reduced expression of GAD67 and GABA transporters in the brain of MDD subjects (90, 93, 94). In addition, genetically modified animals with deletion of specific GABA receptor subunits show depressive phenotypes (95, 96). Furthermore, treatment with various antidepressants (97), electroconvulsive therapy (ECT) (98) and cognitive behavioral therapy (74) tends to restore GABA level in depressed subjects (47). These multiple evidence suggest that impairment in GABAergic transmission plays a significant role in the pathophysiology of depression (99).

## *In vivo* ^1^H-MR Spectroscopy

Proton (^1^H) is the most abundant and sensitive NMR active nucleus, and is an integral part of every neurometabolite. Due to the presence of different functional groups, ^1^H belonging to different molecules or attached to different carbon atoms within the same molecule experiences variation in the electronic environment. This results in differences in ^1^H frequencies, which is commonly known as chemical shift. This parameter is used for the distinction of metabolites by ^1^H-MR spectroscopy without administering any chemical agent.

The neurochemical profile provides valuable information when measured from a well-defined region/volume of the brain. This is measured using localized *in vivo* MR spectroscopy. The localization methods in MR spectroscopy are generally based on magnetic field gradients and radiofrequency pulses. A three dimensional voxel is selected by application of band selective radiofrequency (RF) pulses together with magnetic field gradient along X-, Y- and Z-axes. The most commonly used MR localization methods are described below.

### Image Selected *in vivo* Spectroscopy

This approach employs three frequency selective inversion pulses followed by non-selective excitation of the entire sample in the presence of three orthogonal magnetic field gradients. Image selected *in vivo* spectroscopy (ISIS) achieves complete 3D localization of voxel in eight scans (100).

### Point-Resolved Spectroscopy (PRESS)

This is referred as a double spin-echo localization method, wherein a 90° radiofrequency pulse is followed by two 180° pulses together with magnetic field gradients along three orthogonal axes (101). This produces signals exclusively from the desired volume of interest. Due to complete refocusing of the magnetization, the signal-to-noise ratio (SNR) is relatively higher in point-resolved spectroscopy (PRESS).

### Stimulated Echo Acquisition Mode (STEAM)

It is a single scan localization technique, which involves application of three 90° radiofrequency pulses together with magnetic field gradients along three orthogonal axes. Due to selection of stimulated echo using three slice-selective 90° radiofrequency pulses, stimulated echo acquisition mode (STEAM) provides signals from metabolites at a very short echo time (~5 ms) (102). Furthermore, as all the three pulses are 90° in STEAM, the amount of energy absorbed per mass of tissue is lower in this sequence as compared with PRESS. However, as STEAM focuses only 50% of the magnetization, the SNR of NMR signal in STEAM is 50% of that obtained in PRESS approach.

A combination of these localization methods together with outer volume suppression (103) provides better quality localization, especially when the voxel is relatively small to the entire excited volume. Furthermore, *in vivo* measurements of metabolites whose concentration is in the range of 1–30 μmol/g often encounter huge water signals (55,555 μmol/g), hence requires effective suppression of water for quantification. Various NMR characteristics like relaxation time, scalar coupling, chemical shift and diffusion have been exploited to develop several effective approaches for water suppression. Chemical shift selective (CHESS) (104) and variable pulse powers and optimized relaxation delay (VAPOR) (105) are commonly used approaches for water suppression during *in vivo* ^1^H-MR spectroscopy.

### ^13^C-MR Spectroscopy

**Figure 4:**
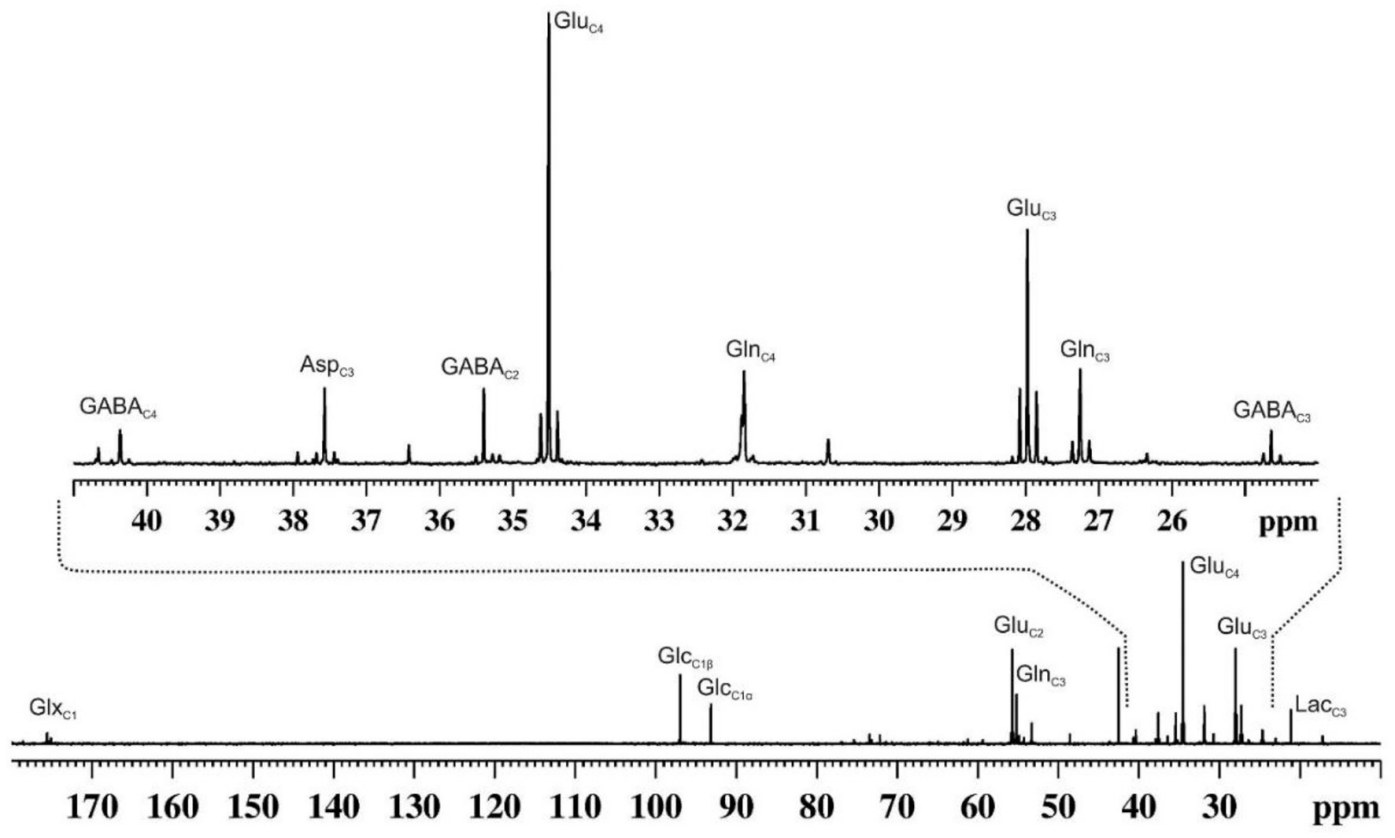
A representative ^13^C NMR spectrum of cortical extract of mouse brain showing labeling of various amino acids from [1,6-^13^C_2_]glucose. Urethane anesthetized mouse was infused with [1,6-^13^C_2_]glucose for 90 min. Brain metabolites were extracted from the cerebral cortex, and ^13^C NMR spectrum of the extract was recorded at 150 MHz NMR spectrometer using power gate decoupling. The spectrum shown in the upper panel is the expansion from 24 to 41 ppm. Asp_C3_, aspartate-C3; GABA_C2_, γ-aminobutyric acid-C2; GABA_C3_, γ-aminobutyric acid-C3; GABA_C4_, γ-aminobutyric acid-C4; Glu_C2_, glutamate-C2; Glu_C3_, glutamate-C3; Glu_C4_, glutamate-C4; Gln_C2_, glutamine-C2; Gln_C3_, glutamine-C3; Gln_C4_, glutamine-C4; Glx_C1_, (glutamate + glutamine)-C1; Glc_C1β_, β-D-glucose-C1; Glc_C1α_, α-D-glucose-C; Lac_C3_, lactate-C3.

^1^H-MRS provides static information for metabolites from a given brain region. In contrast, ^13^C-MRS is very useful in monitoring the flow of labels from ^13^C-labeled substrates to different neurotransmitters such as GABA, glutamate and aspartate, similar to that is used in the tracer approach to evaluate the functional status of tissues and organs (Figure 4). The kinetics of ^13^C labeling of brain amino acids from ^13^C-labeled precursors (glucose/acetate) is useful to estimate the rates of synthesis and catabolism, and thus offer a measurement of neuroenergetics in a given brain region. ^13^C-NMR spectroscopy in the brain has been exploited extensively to understand brain energy metabolism in healthy and different neurological disorders (61).

## Neurometabolites Homeostasis and Metabolism in Depression

As mentioned earlier, the maintenance of neurometabolites homeostasis is critical for the proper functioning of a healthy brain. The changes in the levels of glutamate, GABA, and NAA are often reported under MDD. These are described in details in the following sections.

### Glutamate Homeostasis Under Depression

The ^1^H-MRS method has been used extensively for the assessment of glutamate and other metabolite levels in the brain of depressed subjects and rodent models of depression (Table 1). Reduced level of glutamate has been reported in PFC of mice in different models of depression such as chronic unpredictable mild stress (CUMS) (66), chronic social defeat stress (CSDS) (112, 116), and chronic forced swim stress (CFSS) (122). The decreased glutamate level in PFC has also been reported during the first episode of depression (107, 114). The progress of depression plays a crucial role in abnormalities in glutamate, e.g., chronic or remitted–recurrent MDD subjects showed further reduction in glutamate level in PFC as compared with the first episode depressed subjects (119). Antidepressive medication aids in the restoration of neurometabolite homeostasis to normal level. The unmedicated subjects exhibited lower levels of glutamate plus glutamine (Glx) in the dorsomedial/dorsal anterolateral prefrontal and ventromedial prefrontal cortex as compared with the medicated ones (71). However, there are few inconsistencies in the level of glutamate in depression, as some reports have shown increased glutamate in PFC of the postpartum depressed female subjects (117) and animal model of depression (125).

**Table 1:** Brain glutamate homeostasis in depression.

**S. No.**	**Diagnosis/model**	**Brain region**	**Species**	**Technique**	**Quality index**	**Glu**	**Glx**	**NAA**	**References**
1.	MDD	dACC	Human (H 25, D 51)	MEGAPRESS, 4T	CRI < 19%	↓	–	–	Benson et al. (106)
2.	MDD	vmPFC	Human (H 63, D 31)	PRESS, 3T	CRLB < 30%	↓	↓	–	Draganov et al. 2020 (107)
3.	MDD	RFL	Human (H 32, D 32)	EPSI, 3T	CRLB < 25%	NS	–	↓	Kahl et al. (108)
4.	Depression (CRS)	SSC	Rat (H 8, D 33)	PRESS, 9.4T	CRLB < 15%, NR > 9.5	↓	↓	–	Seewo et al. (109)
5.	Depression (CRS)	NAc	Mice (H 14, D 14)	SPECIAL, 14.1T	CRLB < 20%	↓	–	↓	Cherix et al. (110)
6	BID EU	ACC	Humans (H 80, D 128)	PRESS, 3T	CRLB < 20%, SNR > 10	↑	↑	NS	Soeiro-de-SouZa et al. (111)
7.	Depression (CSDS)	PFC	Mice (H 24, D 25)	*Ex vivo*, ^1^H-[^13^C]-NMR, 14T	–	↓	–	↓	Mishra et al. (112)
8.	MDD	rdPFC	Human (H 33, D 25)	SPECIAL	CRLB < 20%	NS	↓	↓	Jollant et al. (113)
9.	MDD	PFC	Human (H 27, D 22)	PRESS, 3T	CRLB < 10%	↓	↓	–	Shirayama et al. (114)
10.	MDD	HPC	Human (H 38, D 63)	PRESS, 3T	CRLB < 20%	↑	–	–	Hermens et al. (115)
11.	Depression (CSDS)	PFC	Mice (H 15, D 30)	*Ex vivo*, ^1^H-[^13^C]-NMR, 14T	–	↓	–	↓	Veeraiah et al. (116)
12.	PPD	mPFC	Humans (H 12, D 12)	STEAM, 3T	CRLB < 20%	↑	NS	NS	McEwen et al. (117)
13.	Depression (CMS)	PFC & HPC	Rat (H 10, D 10)	PRESS, 7T	CRLB < 20%	↓	↓	↓	Hemanth Kumar et al. (118)
14.	MDD	vmPFC	Humans (H 15, D 45)	PRESS, 3T	CRLB < 30%	↓	–	↓	Portella et al. (119)
15.	MDD	ACC	Humans (H 26, D 23)	PRESS, 3T	CRLB < 20%, SNR > 15	↓	NS	↓	Järnum et al. (120)
16.	MDD	HPC	Humans (H 10, D 18)	PRESS, 3T	–	–	↓	–	Block et al. (121)
17.	Depression (CFSS)	PFC	Mice (H 12, D 12)	*Ex vivo*, ^1^H NMR, 11.7T	–	↓	↓	NS	Li et al. (122)
		HPC				↓	NS	↓	
18.	MDD	dm/da PFC	Humans (H 20, D 20)	PRESS based J editing, 3T	–	–	↓	NS	Hasler et al. (71)
19.	MDD	Subcortical nuclei	Humans (H 21, D 20)	PRESS, 1.5T	–	↓	↓	–	Ajilore et al. (123)
20.	MDD	OCC	Humans (H 38, D 33)	ISIS, J-editing, 2.1T	–	↑	–	–	Sanacora et al. (72)
21.	MDD	ACC	Humans (H 18, D 19)	PRESS, 1.5T	–	↓	↓	NS	Auer et al. (124)

*ACC, anterior cingulate cortex; BID EU, euthymic bipolar I disorder; CFS, chronic forced swim stress; CRS, chronic restraint stress; CUMS, chronic unpredictable mild stress; CSDS, chronic social defeat stress; CRI, Cramer–Rao index; CRLB, Cramer–Rao lower bound; dACC, dorsal anterior cingulate cortex; dm/daPFC, dorsomedial/dorsal anterolateral PFC; EPSI, echo planar spectroscopic imaging; EAP, experimental autoimmune prostitis; HPC, hippocampus; ISIS, image selected in vivo spectroscopy; LD, light deprivation; MDD, major depressive disorder; mPFC, medial prefrontal cortex; MEGA-PRESS, Meshcher–Garwood point-resolved spectroscopy; NAc, nucleus accumbens; NS, no significant change; OCC, occipital cortex; PFC, prefrontal cortex; PPD, postpartum depression; PRESS, point-resolved spectroscopy; rdPFC, right dorsal PFC; RFL, right frontal lobe; SNR, signal-to-noise ratio; SPECIAL, spin echo full intensity acquired localized sequence; SSC, sensorimotor cortex; STEAM, stimulated echo acquisition mode; TRD, treatment resistant depression; UDR, unipolar depression; vmPFC, ventromedial prefrontal region; ↓ depicts decrease, ↑ represents increase. The numbers in the parenthesis under species represent the number of healthy (H) and depressed (D) subject*.

Glutamate level was reported to be decreased together with myo-inositol (a glial marker) and NAA in ACC of depressed subjects (120, 126). Reduced levels of Glx and glutamine have also been reported in the hippocampus of unipolar MDD subjects (121). In accordance with these findings, a reduction in the levels of Glu and NAA have been reported in the hippocampus of chronic mild stress (CMS) (118) and CFSS mouse models of depression (122). In a very recent study, levels of glutamate and glutamine have been reported to be reduced in the sensorimotor cortex of the chronic restraint stressed (CRS) rat model of depression. However, several studies have shown an increase in the level of glutamate in ACC of depressed subjects (106, 111), hippocampus of MDD subjects with alcoholic tendencies (115), and CSDS model of depression in mice.

A meta-analysis of ^1^H-MRS studies involving depressed subjects has revealed a decrease in levels of glutamate and glutamine primarily in ACC including the reduced level of Glx in other brain regions (127). Additionally, a very recent meta-analysis involving a greater number of participants concluded that lower levels of glutamatergic metabolites (glutamate and glutamine) in the medial frontal cortex are linked with the etiology of MDD (128). The reduced level of glutamate in MDD may be due to a lower supply of precursor glutamine by glutamate–glutamine, impaired glucose metabolism, and altered glial activity (64). The impaired functionality of glial cells in depression could lead to a reduction in synaptic glutamate uptake, which may result in elevated extracellular glutamate level that ultimately accelerates neuronal death by glutamate excitotoxicity (129). In fact, reduced expression of excitatory amino acid transporter (EAAT2) and glutamate synthetase (GS) transcripts, which are localized in glia, have been reported in CSDS mouse model of depression (116, 130). These studies support the hypo-glutamatergic hypothesis of depression and suggest that modulation of the glutamatergic system for remission of depression.

### GABA Homeostasis Under Depression

GABAergic system is involved in most psychiatric disorders including major depressive disorder (131), schizophrenia (132), bipolar disorder (133) and autism (134). Several approaches including epigenetics, postmortem studies, and measurement of GABA level in cerebrospinal fluid and plasma have been used to unravel the role of the GABA system in the pathophysiology of psychiatric disorders (131). Lower GABA levels in plasma (135) and cerebrospinal fluid (136) have been reported in depressed subjects. A summary of ^1^H MRS-based measure of GABA level in the depressed subjects as well as in animal models of depression is presented in Table 2. Several studies have shown a lower level of GABA in MDD subjects as compared with healthy controls (131, 154). These include lower GABA concentration in OCC (72, 75, 144), dorsomedial/dorsal anterolateral PFC (71) and ACC of depressed subjects (137, 139, 142, 143, 155). Moreover, a very recent report has shown reduced GABA level in ventromedial PFC (107) of depressed subjects. Additionally, reduced level of GABA has been reported in PFC of chronic stress model of depression in rodents (112, 118). Hence, lower GABA level is often considered as one of the most promising endophenotypes of MDD (156).

**Table 2:** Brain GABA homeostasis in depression.

**S. No.**	**Diagnosis/model**	**Brain region**	**Species**	**Technique**	**Quality index**	**GABA**	**NAA**	**References**
1.	MDD	vmPFC	Human (H 63, D 31)	PRESS, 3T	CRLB < 30%	↑	–	Draganov et al. (107)
2.	MDD	Striatum	Human (H 16, D 20)	J-edited MEGAPRESS, 3T	–	↑	–	Bradley et al. (137)
		ACC				↓	–	
3.	CUMS	HPC	Rat (H 12, D 12)	PRESS, 9.4T	CRLB < 10%	↑	NS	Sekar et al. (138)
4.	MDD	ACC	Human (H 36, D 44)	J-edited, 3T	–	↓	–	Gabbay et al. (139)
5.	MDD	ACC	Human (H 26, D 33)	J-edited, 3T	–	↓	–	Abdallah et al. (140)
6.	MDD	ACC	Humans (H 21, D 20)	J-edited, 3T	–	↓	–	Gabbay et al. (141)
7.	CMS	PFC and HPC	Rat (H 10, D 10)	PRESS, 7T	CRLB < 20%	↓	↓	Hemanth Kumar et al. (118)
8.	MDD	ACC and OCC	Humans (H 24, D 33)	J-edited, 3T	–	↓	–	Price et al. (142)
9.	MDD	pgACC	Humans (H 24, D 19)	2D-JPRESS, 3T	CRLB < 20%	NS	NS	Walter et al. (143)
10.	MDD-R	OCC and ACC	Humans (H 11, D 12)	MEGA-PRESS, 3T	CRLB < 20%	↓	NS	Bhagwagar et al. (144)
11.	MDD	dm/da PFC	Humans (H 20, D 20)	PRESS based J editing, 3T	–	↓	NS	Hasler et al. (71)
12.	PPD	OCC	Humans (H 14, D 9)	J-editing, 2.1T	–	↓	–	Epperson et al. (145)
13.	MDD	OCC	Humans (H 38, D 33)	J-editing, 2.1T	–	↓	–	Sanacora et al. (72)
14.	MDD	OCC	Humans (H 18, D 14)	J-editing, 2.1T	–	↓	–	Sanacora et al. (75)

*ACC, anterior cingulate cortex; CMS, chronic mild stress; CUMS, chronic unpredictable mild stress; CRLB, Cramer–Rao lower bound; dm/daPFC, dorsomedial/dorsal anterolateral PFC; HPC, hippocampus; MDD, major depressive disorder; MDD-R, recovered depression; MEGA-PRESS, Meshcher–Garwood point-resolved spectroscopy; NS, no significant change; OCC, occipital cortex; pgACC, pregenual anterior cingulate cortex; PFC, prefrontal cortex; PPD, postpartum depression; PRESS, point-resolved spectroscopy; SNR, signal-to-noise ratio; vmPFC, ventromedial prefrontal region; ↓ depicts decrease, ↑ represents increase. The numbers in the parenthesis under species represent the number of healthy (H) and depressed (D) subject*.

In contrast to glutamate, whose level is independent of the mood of depressed subjects, the GABA level is state dependent, as its concentration in remitted MDD subjects is similar to healthy controls (40). It has been observed that unmedicated patients had reduced level of GABA in the dorsomedial/dorsal anterolateral PFC as compared with medicated subjects. Additionally, longitudinal ^1^H-MRS studies in MDD subjects have shown restoration of GABA level after electroconvulsive therapy (98), cognitive behavioral therapy (74), treatment with ketamine (150), and selective serotonin reuptake inhibitors (SSRIs) (97). Moreover, ^1^H-MRS measurements have shown lower OCC GABA level in treatment-resistant depressed subjects as compared with non-resistant depressed subjects and healthy volunteers (142, 157).

### N-Acetyl Aspartate Homeostasis Under Depression

NAA is the strongest signal in ^1^H-MRS, and is exclusively localized in neurons. Although the physiological role of NAA in neural function is unclear, it is typically associated with neuronal integrity and mitochondrial health (158). Reduced level of NAA is reported in different brain regions of depressed subjects, including PFC (112, 113), ACC (120, 126), right frontal and parietal lobe (108), and in the hippocampus (122, 159) (Tables 1, 2). A lower level of NAA has also been seen in the hippocampus (122), nucleus accumbens (110) and PFC (112, 116) of rodent models of depression. Reduced levels of NAA along with glutamate suggest decrease in viability of glutamatergic neurons in depression.

### Glutamate and GABA Energy Metabolism in Depression

Positron emission tomography (PET) (160, 161) and ^13^C-MRS are widely used techniques for evaluating brain energy metabolism (162) in humans and rodents. Neurometabolic activities have been investigated using ^13^C-MRS with an administration of ^13^C-labeled substrates (59, 61). As ^13^C-MRS can distinguish labeling of different carbon positions of glutamate, glutamine, GABA and aspartate, it is possible to measure TCA cycle fluxes separately for glutamatergic neurons, GABAergic neurons and astrocytes by appropriate modeling of the ^13^C turnover of neurometabolites (60, 163). Early ^13^C-MRS studies from Shulman et al. have led the foundation of quantitative measurement of rates of neuronal glucose oxidation and neurotransmitter cycling (164, 165). The ^13^C-NMR measurements together with the infusion of ^13^C-labeled substrates in mice (60), rats (163) and human (166) have shown that neuronal mitochondrial TCA cycle in the cerebral cortex contributes ~70–85% of the total energy produced and the remaining (~15–30%) by astroglia. The GABAergic mitochondrial TCA cycle contributes to ~20% of total neuronal TCA cycle in rats (59) and mice cerebral cortex (60). Most of the neuronal energy is used to support the processes associated with glutamate signaling such as postsynaptic glutamate receptors (50%) and action potential (20%) in the cerebral cortex (56, 57). Most importantly, ^13^C-NMR measurements have shown that rates of oxidative glucose metabolism in neurons and neurotransmitter cycling are stoichiometrically (1:1) coupled (165, 167), indicating that energy requirement for cycling of each glutamate molecule is powered by complete oxidation of one molecule of glucose in neurons (168, 169).

There is limited information about brain mitochondrial energetics in depressed subjects. A recent ^13^C-MRS study performed by the Yale Psychiatric group has reported a ~25% reduction in the mitochondrial energy production in glutamatergic neurons in the occipital cortex of depressed subjects (64). However, there was no change in the GABA synthesis rate and glutamate–glutamine neurotransmitter cycling flux. Using CSDS mouse model of depression, we have reported a reduction in the rate of glucose oxidation in glutamatergic and GABAergic neurons in PFC of C57BL6 mice (116). Additionally, glutamate–glutamine cycling was reduced in mice exhibiting depression-like phenotype (112). Moreover, a very recent measurement has revealed decreased glutamatergic (40%) and GABAergic (20%) neurometabolic activity in PFC of CUMS model of depression (66). These alterations were reflected in a large reduction in the rate of neuronal ATP synthesis. Additionally, excitatory and inhibitory synaptic transmissions were reduced by ~40% in these mice. The reduced synaptic transmission in CUMS mice was corroborated by decreased labeling of GABA-C2, Glu-C4, and Gln-C4 from [2-^13^C]acetate (66).

### Effect of Antidepressants on the Glutamatergic and GABAergic Systems

Antidepressants are categorized into different classes: selective serotonin reuptake inhibitors, serotonin–norepinephrine reuptake inhibitors, and selective norepinephrine reuptake inhibitors, which increase the level of synaptic monoamine neurotransmitters by blocking their reuptake in neurons. The antidepressants belonging to the monoamine oxidase inhibitors category increase tissue levels of monoamines by suppressing the activity of corresponding oxidases. These molecules increase synaptic plasticity, activate neurogenesis in the adult hippocampus, and enhance the expression of neurotrophic factors (170, 171). However, despite the increase in brain monoamine level with few doses of conventional antidepressants, the desired outcomes are usually achieved only after several weeks to months of continuous administration (172). Moreover, a significant fraction of subjects, commonly referred to as treatment-resistant, do not respond to these antidepressants despite the use of various therapeutic strategies (173).

Interestingly, a single subanesthetic dose of ketamine, a non-competitive NMDA channel blocker, produces rapid antidepressant actions within hours of administration, and the effects last for several days (150, 174) (Table 3). Although, the precise mechanism of ketamine action is elusive, various studies have reported that acute intervention with a low dose of ketamine increases glutamate efflux in PFC of mice and rats (112, 175, 176). These studies led to hypothesize that partial antagonism of NMDA receptor by a subanesthetic dose of ketamine may induce antidepressive effects by increasing neurotransmission and neurometabolism in PFC (175). Moreover, the antidepressive effects of ketamine could be related to the selective impact on GABAergic interneurons. Ketamine blocks the NMDA receptors of GABA interneurons, thus suppresses their ability to inhibit pyramidal neurons, thereby induces cortical excitation (11, 177).

**Table 3:** Impact of ketamine on neurometabolites homeostasis in depression.

**S. No.**	**Species**	**Brain region**	**Sample size**	**Dose**	**Technique**	**Quality index**	**Glu**	**Glx**	**GABA**	**References**
1.	Human (MDD)	pgACC	HP: 12, HK: 11 pavee DP: 16, DK: 18	0.5 mg/kg (iv) for 40 min	PRESS, 7T	SNR > 150	NS	–	–	Evans et al. (146)
2.	Human (HV)	ACC	HP: 16, HK: 31	0.23 mg/kg (iv) in 1 h	PRESS, 3T	–	–	↑	–	Javitt et al. (147)
3.	Humans (HV)	pgACC	HP: 14, HK: 12	0.5 mg/kg (iv) for 40 min	STEAM, 7T	CRLB < 20%	↓	–	–	Li M. et al. (148)
4.	Human (HV)	HPC	HP: 12, HK: 15	0.27 mg/kg (iv)	PRESS, 3T	CRLB < 20%	–	↑	–	Kraguljac et al. (149)
5.	Human (MDD)	mPFC	DK: 11	0.5 mg/kg (iv) for 40 min	J-editing, 3T	–	–	↑	↑	Milak et al. (150)
6.	Rats (Social isolation)	ACC	HP: 8, HK: 8	25 mg/kg (ip)	PRESS, 7T	CRLB < 25%	NS	–	↓	Napolitano et al. (151)
7.	Rat (CUS)	ACC	HP: 5, HK: 6 pavee DP: 6, DK: 7	40 mg/kg (ip)	*Ex vivo* CPMG, 11.7T	CRLB < 20%	NS	NS	↓	Perrine et al. (152)
8.	Rat (H)	PFC	HP: 12, HK: 12	30 mg/kg (sc) for 6 days	PRESS, 4.7T	CRLB < 30%	↑	–	–	Kim et al. (153)

*ACC, anterior cingulate cortex; CPMG, Car–Purcell–Meiboom–Gill; CRLB, Cramer–Rao lower bound; CUS, chronic unpredictable stress; DK, depressed subject with ketamine; DP, depressed subject with placebo; HK, healthy subject with ketamine; HP, healthy subject with placebo; HPC, hippocampus; HV, Healthy volunteer; ip, intraperitoneal; iv, intravenous; MDD, major depressive disorder; NS, no significant change; PFC, prefrontal cortex; pgACC, pregenual anterior cingulate cortex; PRESS, point-resolved spectroscopy; SNR, signal-to-noise ratio; sc, subcutaneous; STEAM, stimulated echo acquisition mode; ↓ depicts decrease, ↑ represents increase*.

A very recent pilot study has evaluated the impact of intravenous ketamine administration on neurotransmitter levels in the medial prefrontal cortex (mPFC) of MDD subjects (150). GABA/water and Glx/water peaked ~38% above baseline within 30 min of ketamine infusion (150) (Table 3). However, the majority of the studies reported insignificant changes in GABA and glutamate levels following ketamine treatment (178, 179). As mentioned above, the antidepressant effects of ketamine could be related to its impact on neurotransmitter cycling, oxidative energy metabolism, and neuronal–astroglial coupling. Very recently, we have shown that the subanesthetic dose of ketamine (10 mg/kg, intraperitoneal) increases ^13^C labeling of glutamate, GABA and glutamine from glucose and acetate in PFC of CSDS mice. These findings indicate that ketamine normalizes the neurometabolic activity of glutamatergic and GABAergic neurons along with astrocytes in depression (112, 175). Moreover, recent studies with ketamine in MDD subjects indicated an increase in the rate of glutamate–glutamine neurotransmitter cycling without any change in oxidative energy production in neurons (180, 181).

## Outlook

The homeostasis of tissue glutamate and GABA plays important role in neural activity. The GABAergic neurons are known to control the dopaminergic reward circuitry in the VTA (182, 183). Alteration in the GABAergic neurotransmission with defective GABA_A_ receptor subunits (94, 95, 184) and GAD67 (90, 93) have been reported in depressed subjects. Moreover, modulation of GABAergic activity in mice using genetic and optogenetic approaches leads to anhedonia and neophobia, which are characteristics of depressive disorder (185, 186). The reduced regulatory inhibition on principal neurons may lead to the excessive release of excitatory neurotransmitters in the synapse. The elevated glutamate level in the synaptic cleft stimulates prolonged and excessive activation of NMDA receptors (187). This increased neural activity ultimately leads to atrophy of glutamatergic neurons by excitotoxicity. A homeostatic reduction in glutamate receptors and functional impairment of glutamatergic synapses in the hippocampus and medial prefrontal cortex have been reported in γ2-subunit of GABA_A_ receptor knockout mice, which exhibit a modest defect in GABAergic transmission (188).

^1^H-MRS measures combined intracellular and extracellular glutamate and GABA pool in neurons and glia. The intracellular neurotransmitter pool dominates excessively with the extracellular (2,000–5,000:1) (189). Therefore, ^1^H-MRS measured changes in the levels of glutamate and GABA may not reflect the abnormalities in synaptic concentration and *vice versa*. Hence, the findings of ^1^H-MRS studies should be interpreted with great caution. ^1^H- and ^13^C-NMR measurements have revealed several vital information about depression. Limited measurements based on ^13^C-NMR spectroscopy together with administration of ^13^C precursor have suggested a reduced rate of glucose oxidation, neuronal and astroglial metabolic activity, and altered neurotransmitter trafficking in the prefrontal cortex in depression. However, there are some inconsistencies in the literature, which may be attributed to differences in the disease severity, age, gender, comorbidity, the investigated brain regions, the status and duration of medications in subjects. Hence, there is a further need for comprehensive large-scale collaborative analysis about neurotransmitter homeostasis and their energetics to better understand the etiology of depression similar to that proposed by the ENIGMA consortium for genetic and neuroimaging data.

## Author Contributions

AS: literature survey, preparation of figure, manuscript writing, and editing. NS: preparation of figure, manuscript writing, and editing. AP: conception of the idea, preparation of figures, manuscript writing and editing, supervised and directed the overall project. All authors contributed to the article and approved the submitted version.

## Conflict of Interest

The authors declare that the research was conducted in the absence of any commercial or financial relationships that could be construed as a potential conflict of interest.
